# Long lasting dysautonomia due to botulinum toxin B poisoning: clinical- laboratory follow up and difficulties in initial diagnosis

**DOI:** 10.1186/1756-0500-6-438

**Published:** 2013-10-30

**Authors:** Anna Potulska-Chromik, Beata Zakrzewska-Pniewska, Elżbieta Szmidt-Sałkowska, Jacek Lewandowski, Maciej Siński, Witold Przyjałkowski, Anna Kostera-Pruszczyk

**Affiliations:** 1 Department of Neurology, Medical University of Warsaw, Banacha 1a Street, 02-097 Warsaw, Poland; 2 Department of Internal Medicine, Hypertension and Vascular Diseases, Medical University of Warsaw, Warsaw, Poland; 3 Institute of Infectious and Parasitic Diseases, Medical University of Warsaw, Warsaw, Poland

**Keywords:** Clostridium botulinum B, Botulism, Dysautonomia

## Abstract

**Background:**

Botulism is an acute form of poisoning caused by one of four types (A, B, E, F) toxins produced by Clostridium botulinum, ananaerobic, spore forming bacillus. Usually diagnosis of botulism is considered in patients with predominant motor symptoms: muscle weakness with intact sensation and preserved mental function.

**Case presentation:**

We report a case of 56-year-old Caucasian female with a history of arterial hypertension, who presented with acute respiratory failure and bilateral ptosis misdiagnosed as brainstem ischemia. She had severe external and internal ophtalmoplegia, and autonomic dysfunction with neither motor nor sensory symptoms from upper and lower limbs. Diagnosis of botulinum toxin poisoning was made and confirmed by serum antibody testing in the mouse inoculation test.

**Conclusions:**

Ophtalmoplegia, autonomic dysfunction and respiratory failure can be caused by botulism. Early treatment and intensive care is essential for survival and recovery. The electrophysiological tests are crucial to correct and rapid diagnosis. Botulism (especially type B) should be considered in any case of acute or predominant isolated autonomic dysfunction.

## Background

Clostridium botulinum neurotoxin is a rare cause of a life-threatening disease usually manifesting as descending symmetrical paralysis without sensory findings [1,2]. Currently cases of botulism are still reported e.g. after consumption of canned food or honey ingestion. Below we present difficulties in diagnosing botulism with autonomic involvement.

## Case presentation

A 56-year-old woman with acute respiratory failure and bilateral ptosis, with a past medical history of arterial hypertension was referred by regional hospital to Intensive Care Unit at the Department of Neurology. On admission to the previous hospital she presented with nausea and vomiting. One day after the onset of the disease she experienced dysphagia and blurred vision. She was first diagnosed with a brainstem stroke but CT of her brain was normal. 24 hours later bilateral ptosis and dyspnea developed. On admission to Intensive Neurological Care Unit she had sever dysarthria, her hypoventilation and hypoxia progressed to respiratory failure. Detailed neurological examination revealed external and internal ophtalmoplegia (equal unreactive pupils), deep tendon reflexes were relatively brisk. No other abnormalities, including limbs muscles paralysis, were observed. The patient was intubated and mechanical ventilation was commenced. Both brain CT and MRI scans were normal. Serum electrolytes, glucose and blood count were normal. CSF parameters were within normal limits. Nerve conduction studies showed slightly decreased amplitude of CMAP in the median nerve, with normal conduction velocities and normal conduction parameters in other motor nerves. Sensory NCV studies were within normal limits. Electrophysiological repetitive stimulation test of ulnar and facial nerve was normal. Other electrophysiological tests, such as SFEMG and SSR, were not done because the patient tolerated examination very poorly. Although repetitive stimulation test was negative, after detailed anamnesis with her family presumptive diagnosis of botulinum toxin poisoning was made and trivalent ABE antitoxin was administered on the day of her admission to our department. Serum sample was sent for botulism mouse inoculation test. The results reported 4 days later were positive for type B Clostridium botulinum toxin. The patient gradually improved and regained strength but remained on mechanical ventilation for 7 days. Finally, a possible source of botulism was established: the patient was only person in her family who had eaten a jar of pate one day prior to her admission to hospital. The pate was not available for toxicology testing.

### Results of clinical and laboratory follow up

Four months after the onset of the disease the patient still experienced episodic palpitation and chronic fatigue. On neurological examination only slow reaction of pupils to the light and convergence was found. Detailed cardiovascular and autonomic function tests are presented in Table 1. All tests showed significant but not life threatening signs of autonomic system involvement.

**Table 1:** Results of the cardiovascular and electrophysiological study performed > four months after the onset of symptoms

**Name of the test**	**Results**
Holter ECG	Sinus rhythm, mean HR 85/min with numerous single extrasystole (over 3,000 excitations), 20 episodes of ventricular tachycardia
Heart USG	Decreased global heart muscle contractility with a decline of ejection fraction. No akinesis or other features characteristic of ischemia
Sympathetic skin response	No responses to auditory or electrical stimulation
RR interval variation test (RRIV)	Abnormal, with HRV decreased at rest-ranged from 3.99-5.78 as well as during deep breathing (5.91%), without.an increase in HRV on hyperventilation
SFEMG from right external digitorum communis muscle (SF-EMG)	Mean jitter value increased to 53 us (norm 32+/−5.7), in 4 pairs above 55 useconds

## Discussion

Our patient presented with predominant acute autonomic dysfunction and respiratory failure due to ingestion of Clostridium botulinum toxin type B. The neurotoxin is known to be a cause of a variety of autonomic symptoms [1,2]. The initial gastrointestinal symptoms are typical but not specific for botulism. Blurred vision, diplopia, opthalmoplegia, which had occurred in our patient 12 hours after onset of the disease, were misdiagnosed as a brainstem stroke. However, autonomic nervous system dysfunction was manifested at the same time. The presence of autonomic dysfunction should be differentiated from cholinergic dysautonomia, a rare condition mainly affecting children and young adults following viral infection, with relative preservation of sensory and motor function [3]. Our patient presented with respiratory failure caused by respiratory muscle weakness, which is not observed in cholinergic dysautonomia. Usually, the diagnosis of botulism is considered in patients with predominant motor symptoms: descending muscle weakness with intact sensation and mental function [4-6], but also predominant cranial symptoms of botulism were recently reported [7,8]. In our patient bulbar and respiratory muscle dysfunction occurred. Differential diagnosis included neuromuscular disorders such as Guillain-Barre syndrome, or its variant Miller–Fisher syndrome [4,6,7]. Preservation of sensation and deep tendon reflexes, normal nerve conduction parameters and normal CSF protein level do not support diagnosis of inflammatory polyneuropathy. In most cases of botulism nerve conduction studies demonstrate low amplitude CMAPs with normal conduction velocities and sensory nerve action potentials [9,10]. This, and repetitive nerve stimulation test results may be normal in patients with predominant autonomic dysfunction without limb weakness. SFEMG and SSR seem to be more sensitive studies of botulism and should be performed in patients with suspected botulism [9,10]. These tests could also be used in monitoring dysautonomia.

## Conclusions

Botulism should be considered in patients with acute or subacute autonomic dysfunction. Autonomic function should be evaluated and monitored in every patient with botulinum intoxication. Dysautonomia, even subclinical, may be a potential life threatening consequence of botulism.

## Consent

Written informed consent was obtained from the patient for publication of this case report and accompanying images.

## Abbreviations

CT: Computed tomography; MRI: Magnetic resonance imaging; CSF: Cerebrospinal fluid; CMAP: Compound action potential; NCV: Nerve conduction velocity; SFEMG: Single fiber; EMG: Single fiber electromyography; SSR: Sympathetic skin response; RRIV: R, R interval; HRV: Heart rate variation; ECG: Electrocardiogram; USG: Ultrasonography; HR: Heart rate; MSNA: Muscle sympathetic nerve activity.

## Competing interests

The authors declare that they have no competing interests.

## Authors’ contributions

APCH diagnosed the patient, analyzed and interpreted the patient data including biochemistry, EMG and MRI results and was a major contributor in writing the manuscript. BZP performed and interpreted the sympathetic skin response as well as RRIV examination and she has a contribution in writing the manuscript. ESS performed EMG examination and she was the contributor in data interpretation and writing the manuscript. JL performed microneurography and interpreted the patient data regarding cardiovascular system, including patients laboratory follow up. MS performed heart USG, interpreted Holter ECG and analyzed results of the examination of cardiovascular system, including laboratory follow up. WP help in diagnosis of the patient, interpreted results of the mouse inoculation test, under his care patient obtained trivalent ABE antitoxin. AKP analyzed and summarized all electrophysiological results including SFEMG, microneurography, SSR and RRIV, and was a major contributor in writing the manuscript. All authors read and approved the final manuscript.
